# Examining Brain-Cognition Effects of Ginkgo Biloba Extract: Brain Activation in the Left Temporal and Left Prefrontal Cortex in an Object Working Memory Task

**DOI:** 10.1155/2011/164139

**Published:** 2011-08-18

**Authors:** R. B. Silberstein, A. Pipingas, J. Song, D. A. Camfield, P. J. Nathan, C. Stough

**Affiliations:** ^1^Centre for Human Psychopharmacology, Swinburne University, P.O. Box 218, Hawthorn, Victoria, Australia; ^2^Clinical Unit Cambridge, GlaxoSmithKline Pharmaceuticals, Cambridge, UK

## Abstract

Ginkgo Biloba extract (GBE) is increasingly used to alleviate symptoms of age related cognitive impairment, with preclinical evidence pointing to a pro-cholinergic effect. While a number of behavioral studies have reported improvements to working memory (WM) associated with GBE, electrophysiological studies of GBE have typically been limited to recordings during a resting state. The current study investigated the chronic effects of GBE on steady state visually evoked potential (SSVEP) topography in nineteen healthy middle-aged (50-61 year old) male participants whilst completing an object WM task. A randomized double-blind crossover design was employed in which participants were allocated to receive 14 days GBE and 14 days placebo in random order. For both groups, SSVEP was recorded from 64 scalp electrode sites during the completion of an object WM task both pre- and 14 days post-treatment. GBE was found to improve behavioural performance on the WM task. GBE was also found to increase the SSVEP amplitude at occipital and frontal sites and increase SSVEP latency at left temporal and left frontal sites during the hold component of the WM task. These SSVEP changes associated with GBE may represent more efficient processing during WM task completion.

## 1. Introduction

Ginkgo biloba extract (GBE) from dried leaves of the tree Ginkgo biloba is increasingly used in Europe (especially Germany) and the United States of America to alleviate symptoms associated with age-related cognitive impairment such as age-related amnesic condition, vascular dementia and dementia of the Alzheimer's type [1–6]. Recent studies appear to indicate that GBE (120–600 mg) moderately enhances a number of cognitive processes in healthy young individuals as well as those suffering from age related cognitive impairment [7–17]. 

A number of trials of GBE in the treatment and prevention of Alzheimer's disease (AD) have also demonstrated significant improvements to cognition [18]; however, its efficacy is not without contention. A large American multicentre study by DeKosky and colleagues [19] reported no significant reduction in the incidence of AD in 3069 participants, with a median followup of 6.1 years. However, recently released top line findings from a large-scale European study with 2584 elderly individuals (GuidAge; [20]) revealed significant delays in conversion to AD in patients treated for at least 4 years with GBE (IPSEN [21]). 

A criticism of large-scale studies of GBE in dementia has been that they show a preference for the measurement of global rather than specific effects and for this reason leave unanswered the question of the primary effect of GBE on cognition [22]. One such cognitive domain that GBE has shown to improve across a number of studies is Working Memory (WM) [10, 15, 23, 24]. A previous study from our laboratory [15] revealed that 30-day administration of 120 mg/day GBE in young volunteers resulted in significant improvements in both digit span backwards and working memory speed. In a review of 29 randomized clinical trials examining the cognitive effects of GBE, Kaschel [22] concluded that as many as 20.7% of WM tests in chronic GBE trials had yielded significant results. 

The mechanism by which GBE achieves improvements in WM is unclear although it is unlikely to involve a single process. The active compounds of GBE are primarily flavonol glycosides (24%) and terpene compounds such as ginkgolides and bilobalide (6%) with smaller amounts of proanthocyanidins [25]. The compounds have been shown to possess potent free radical scavenging and antioxidant properties that may play an important role in the neuroprotective properties of GBE [5, 25, 26]. GBE also influences a number of neurotransmitter systems that are considered critical in cognition [26, 27]. In particular, GBE is thought to enhance cholinergic processes in various cortical regions [5, 28]. Animal in vitro studies indicate that GBE increases acetyl-choline (ACh) release in hippocampal synaptosomes [29]. Animal in vivo studies indicate that GBE improves performance on behavioural measures of spatial working memory [30], attenuates the amnesia induced by scopolamine [31], and increases the density of hippocampal muscarinic receptors [32]. 

Modulation of the cholinergic system is known to influence cognitive processes, especially WM [33]. Increases in cholinergic transmission are known to enhance WM performance [34–37], while reductions in cholinergic transmission compromise performance on WM tasks [38, 39]. Functional brain imaging studies have revealed that modulation of the cholinergic system has different effects on specific neural subsystems mediating WM. In a faces WM task, Physostigmine, an acetylcholinesterase inhibitor, improved performance. These improvements were associated with increased cerebral blood flow in the visual cortex and decreased blood flow at right prefrontal cortex, left temporal cortex, and left hippocampus/parahippocampal gyrus [34, 35]. 

There have been a number of studies to investigate the effects of GBE supplementation on brain activity; however, the majority of these have only examined the effects of GBE on EEG frequency during a resting state. Itil and colleagues [40] reported dose-dependent increases in occipital EEG alpha band activity during resting eyes closed following single doses of 40, 120, and 240 mg GBE. A more recent study by Kennedy and colleagues [41] with participants under the age of 40 yrs reported that “eyes closed” EEG power in both theta and beta bands was reduced across frontal regions at 4 hours after a single 360 mg dose of GBE. In relation to the chronic effects of GBE on EEG frequency, Hofferberth [42] reported a significant reduction in theta waveband activity associated with 3 months GBE at 240 mg/day in elderly patients with AD. The finding of a decrease in theta power in conjunction with an increase in alpha power associated with GBE supplementation is in line with the frequency profile that has been found to be associated with good cognitive and memory performance [43]. 

In addition to the analysis of EEG frequency at rest, another electrophysiological finding that has been reported in relation to GBE supplementation is a reduction in P300 latency. In one such study, Semlitsch and colleagues [44] reported reductions in P300 latencies following both a single 120 mg dose of GBE as well as following 57 days of GBE at 120 mg/day in elderly patients with age-associated memory impairment. The authors suggested that the decrease in P300 latency associated with GBE may be a reflection of shorter stimulus evaluation time. In addition to electrophysiological studies of the effects of GBE on the brain, Santos and colleagues [45] utilized single photon emission computed tomography (SPECT) to examine the neurophysiological effects of 8 months of 80 mg/day GBE supplementation versus placebo in elderly healthy males. Santos and colleagues [45] reported that the group receiving GBE displayed increased cerebral perfusion in several areas including bilateral frontal, bilateral parietal, right-frontal parietal, left temporal, and right occipital brain regions. Similar improvements to cerebral perfusion have also been reported following GBE treatment for brain circulation insufficiency [46]. 

In order to better understand the neurophysiology behind improvements in working memory ability brought about by GBE, it is pertinent to examine changes in brain electrical activity whilst completing a WM task which has previously been shown to be improved after GBE administration. In the present study, we sought to examine the effects of GBE on steady-state visually evoked potential (SSVEP) topography in healthy middle-aged human subjects, while they performed an object WM task. Previous studies have revealed characteristic changes in the 13 Hz SSVEP (termed steady state topography; SST) during a delayed-match-to-sample object WM task. Specifically, during the intake or perceptual component, we have previously observed decreased SSVEP amplitude and decreased SSVEP latency at occipital sites. During the hold component, we have observed an SSVEP amplitude increase at occipital and medial prefrontal sites and decreased SSVEP latency at prefrontal sites [47, 48]. In line with studies of WM that have utilized functional brain imaging [49, 50], these latency changes have been interpreted in terms of increased excitation of occipital cortex during the intake component and increased prefrontal excitation during the hold component of the WM task [48]. If GBE effects are mediated by cholinergic processes and the effects are similar to those of physostigmine as reported by Furey and colleagues [34, 35], then it would be expected that the effects of chronic GBE treatment in an object WM task will be to increase SSVEP latency (corresponding to reduced excitation or increased inhibition) at temporal and prefrontal sites. In relation to the behavioural effects of GBE, we hypothesized that there would be a significant improvement in both the speed and accuracy of performance in an object WM task following chronic GBE treatment.

## 2. Methods

### 2.1. Participants

Nineteen right handed individuals (10 males) aged between 50 and 61 years (mean 54.9 ± SD 3.1) participated in the study. Inclusion criteria for this study were that the subjects be right handed as determined by the Edinburgh Handedness Inventory and have normal uncorrected vision. Exclusion criteria included past history of head injury requiring hospitalization, intellectual developmental disability, neurological or psychiatric illness, epilepsy, and/or a past or current history of substance abuse. The study was approved by the Human Research Ethics Committee of Swinburne University of Technology, and all participants provided written informed consent.

### 2.2. Procedure

A randomized double-blind crossover design was employed in which participants were randomly allocated to two groups (Group A or B). Participants in Group A were administered GBE for 14-day followed by a 14 days washout period (no tablets). The washout period was then followed by 14 days of placebo administration. SST recordings were conducted at baseline (before-GBE), 14 days following GBE (after-GBE), baseline at the end of the washout period (before-placebo), and 14 days following placebo treatment (after-placebo). The GBE and placebo treatment periods were reversed for Group B. The daily dosage was two tablets of Blackmore's Ginkgoforte (2 × 40 mg) or 2 identical placebo tablets. Each tablet contained Ginkgo biloba extract equivalent to 2 g dry leaf and was standardized to contain 10.7 mg ginkgo flavonol glycosides and 2.7 mg ginkgolides and bilobalide.

#### 2.2.1. Cognitive Tasks

During each of the four SST recording sessions, participants performed three cognitive activation tasks: an object WM task, an abstract shape recognition task, and the continuous performance task, a task designed to tap sustained attention. This study reports on the WM findings, findings on the recognition memory, and attention activation tasks will be presented elsewhere.

Participants performed an object WM task where each trial required them to hold one or two irregular polygons in WM. Irregular polygons were selected to minimize the chance of participants using verbal strategies in the task [51]. Each trial was preceded by a 1.5 sec interval where participants fixated on a cross located at the center on a blank screen. This was followed by a 1.1 sec interval where the target, comprising one or two irregular polygons, appeared on the screen. Immediately following the target, a mask consisting of a circular annulus of radius 3° appeared on the screen for 0.2 sec. During the subsequent 3.0-second hold period, the screen was blank except for a small cross in the centre of the screen that acted as a fixation point. Subjects were then presented with an irregular object (the probe) and required to indicate whether the object matched one of the polygons prior to the hold period. A button push with the right hand indicated a match, while a nonmatch was indicated by a left button push. Each trial lasted 12 seconds and subjects performed 20 trials in a 4-minute block. All subjects undertook two blocks of the task. Reaction time for each trial was recorded to an accuracy of 1 msec.

**Control Task**

Participants also performed a control task identical in structure to the WM task except that the “hold” interval was reduced from 3.0 sec to 0.25 sec, and the pretrial blank screen was increased in duration from 1.5 sec to 4.5 sec.

**Stimuli**

Each of the polygons subtended a horizontal and vertical angle of approximately 1.0° when viewed by the subjects from a fixed distance of 1.34 m. Polygons and circles had a luminance of 13.0 Cd/m^2^ against the video monitor background of 1.2 Cd/m^2^. All polygons were located on an imaginary circle of radius 3.0° centered on the fixation cross. The stimulus used to evoke the SSVEP was a 13 Hz sinusoidal flicker subtending a horizontal angle of 160° and a vertical angle of 90°. The modulation depth of the stimulus when viewed against the background was 45%. A set of goggles, which permitted the sinusoidal flicker to be superimposed on the viewing field, was used to present the stimulus [52]. The goggles comprised two sets of light-emitting diode (LED) arrays viewed through half-silvered mirrors. The light intensity generated by the LED arrays was controlled by a 13 Hz sinusoidal voltage waveform, and the nonlinearity between voltage input and light intensity was less than 0.5%.

#### 2.2.2. Recording

Brain electrical activity was recorded from 64 scalp electrode sites, which included all international 10–20 positions as well as additional sites, located midway between 10 and 20 locations. The specific locations of the recording sites have been previously described [53]. The average potential of both earlobes served as a reference, and a nose electrode served as ground. Brain electrical activity was amplified and band-pass filtered (3 dB down at 0.1 Hz and 80 Hz) prior to digitization to 16-bit accuracy at a rate of 500 Hz.

#### 2.2.3. Signal Processing

The major features of the signal processing have already been described [53, 54]. Briefly, the SSVEP was determined from the 13 Hz Fourier coefficients evaluated over 10 stimulus cycles at the stimulus frequency of 13 Hz, thus yielding a temporal resolution of 0.77 sec. The 10-cycle evaluation period is shifted 1 stimulus cycle, and the coefficients are recalculated for this overlapping period. This process was continued until the entire period of activity for each block was analyzed. An identical procedure was applied to data recorded from all 64 electrodes.

To assess the changes in the SSVEP associated with different components of the cognitive tasks, the following procedure was employed. For all trials, 10 sec epochs of SSVEP real and imaginary components commencing 5.0 sec before the start of the “hold” component were averaged, for all correct responses. For each subject and each electrode site, the mean SSVEP amplitude and phase (expressed as a single complex number) was determined from these 10 sec SSVEP epochs of the control task undertaken in the postplacebo recording session. This yielded 64 measures of the mean SSVEP amplitude and phase (one for each electrode) during the low-demand attention task for each subject. The 64 amplitude measures were then averaged to yield a mean SSVEP amplitude for each subject, termed the normalization factor (NF). Pooled effects were examined by weighted averaging of the mean SSVEP time series for object WM trials for all 19 subjects. The weighted averaging procedure involves normalization of the SSVEP amplitude time series prior to averaging or pooling across subjects. This is necessitated by the large intersubject variation in the SSVEP amplitude [52]. Normalization was achieved by dividing the mean SSVEP amplitude time series for the WM trials for each subject by the appropriate NF. The pooled SSVEP amplitude was then represented as a multiple of the normalization factor. Variations in the SSVEP phase were expressed in terms of latency variations.

#### 2.2.4. Artifact Detection and Compensation

A specific advantage of the SSVEP is its relative noise and artifact insensitivity [53, 55]. This is a consequence of the fact that signal power of artifacts such as the electro-oculogram (EOG) and eye blinks is located primarily at low frequencies and is negligible above 8 Hz [56], while muscle electrical activity is distributed over a range of frequencies [55]. By contrast, the SSVEP power is concentrated almost exclusively at the stimulus frequency, that is, 13 Hz and its harmonics [55]. The signal processing technique we have used to extract the SSVEP is only sensitive to a narrow frequency band centered on the stimulus frequency and is thus less influenced by artifact frequency components that differ from the stimulus frequency. The relative insensitivity of the SSVEP to common artifacts permits one to relax the rejection criteria for artifact contamination that are normally employed when evaluating EEG power spectra. For each subject, the mean SSVEP time series for the WM task were visually inspected, and any recording site that was identified as a failure was replaced by the mean of its 3 nearest neighboring recording sites.

#### 2.2.5. Mapping and Statistical Considerations

Topographic maps illustrating the differences in SSVEP latency and amplitude between the pre-GBE and post-GBE conditions were produced using a spherical spline interpolation procedure [57]. Statistical probability mapping (SPM; [58]) based on Hotelling's *T* ^2^ measures was based on multiple bivariate paired *T* ^2^ test, comparing the SSVEP time series during the post-GBE session with the equivalent point in time for the post-placebo session. Those regions of the SPM maps where *T* (the square root of Hotelling's *T* ^2^) equals 2.88, 3.19, and 3.92 are represented by 3 iso-*T* contours and correspond to single comparison *P* values of 1%, 0.5%, and 0.1%, respectively. 

To account for the multiple comparison undertaken, the Bonferroni correction was used, based on the spatial dimensionality of our data [54]. While there are 64 comparisons undertaken for each point in time (one for each electrode), these comparisons are not independent, as the EEG at neighboring electrodes is highly correlated. The spatial dimensionality of the data is thus not 64, the number of scalp recording sites, but a smaller number that takes into account the correlation between neighboring sites. Spatial principal components analysis (SPCA) of scalp EEG and scalp SSVEP suggests that no more than 5 factors are required to account for at least 95% of the spatial variance [59, 60]. For a single set of comparisons based on the 64 scalp recording sites, this suggests a Bonferroni adjusted *P* value of 0.05/5, that is, 1%. If we consider the 2 Hotelling's *T* maps presented in the Results section as 2 independent comparisons, then an additional application of the Bonferroni correction is required and the adjusted *P* value of 1% needs to be divided by 2 to yield the final adjusted *P* value of 0.5% as the experiment-wise probability of 5% for incorrectly rejecting the null hypothesis.

## 3. Results and Discussion

### 3.1. Behavioral Results

We observed an increase in accuracy from 71.7% in the postplacebo condition to 76.8% in the post-GBE condition (paired *t*-test, *t* = 2.34; *df* = 18; *P* = 0.037). We also observed a non-significant reduction in reaction time from 1029 msec in the postplacebo condition to 1012 msec in the post-GBE condition (paired *t*-test, *t* = 0.74; *P* = 0.46). We observed a modest increase in WM accuracy immediately after 14-day treatment with GBE. Such an increase is consistent with other findings that point to an improvement in WM [10, 15, 24]. While a trend towards faster reaction times for the WM task was also observed, this result did not reach statistical significance.

### 3.2. Brain Electrical Activity

#### 3.2.1. Task-Related Effects

**Figure 1:**
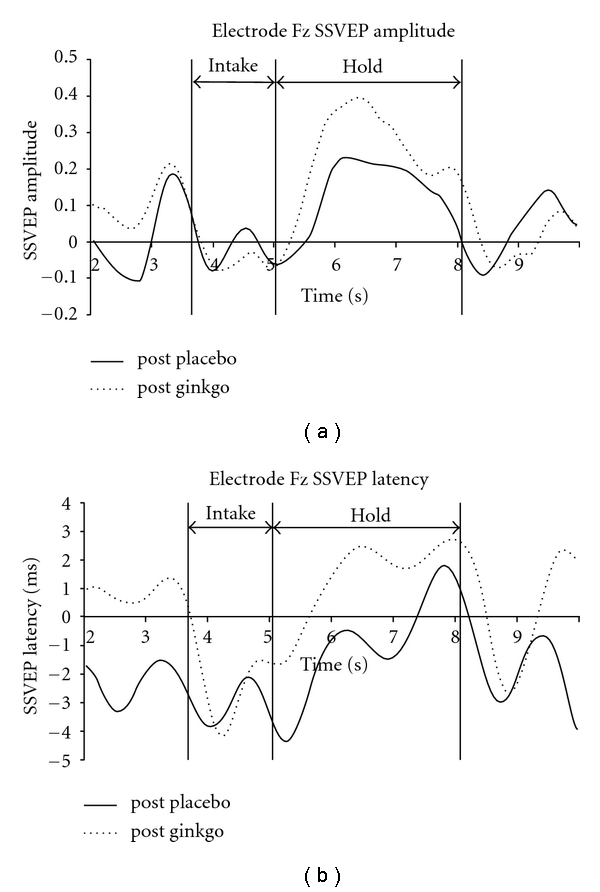
(a) SSVEP amplitude at the midline prefrontal electrode location Fz. SSVEP amplitude is referenced to the mean SSVEP amplitude averaged over the 10 sec epoch for the post-placebo control task. Note the increased SSVEP amplitude increase during the “hold” component compared with the “intake” component. The GBE related increase in SSVEP amplitude is most prominent during the hold component. (b) SSVEP latency at the midline prefrontal electrode location Fz. SSVEP latency is referenced to the mean SSVEP latency averaged over the 10 sec epoch for the post-placebo control task. In the post-placebo condition, the SSVEP latency reduction during the intake component becomes less prominent during the hold component and reverses to a latency increase at the end of the hold component. Note the SSVEP latency increase associated with the GBE.

**Figure 2:**
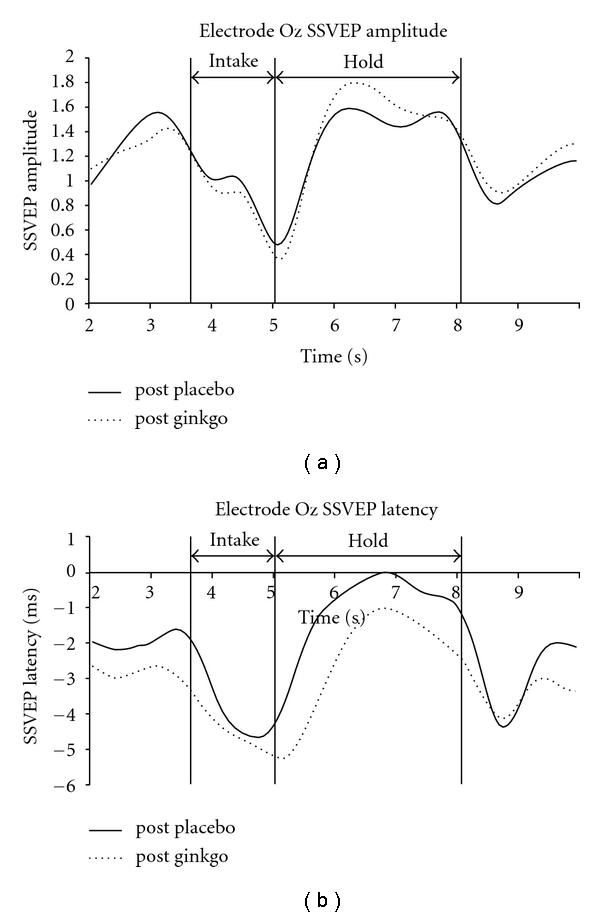
(a) SSVEP amplitude at the midline occipital electrode location Oz. SSVEP amplitude is referenced to the mean SSVEP amplitude averaged over the 10 sec epoch for the post-placebo control task. Note that greater amplitude increase during the hold period for the GBE condition. (b) SSVEP latency at the midline occipital electrode location Oz. SSVEP latency is referenced to the mean SSVEP amplitude averaged over the 10 sec epoch for the post-placebo control task. Note the sustained SSVEP latency reduction in the GBE condition.

**Figure 3:**
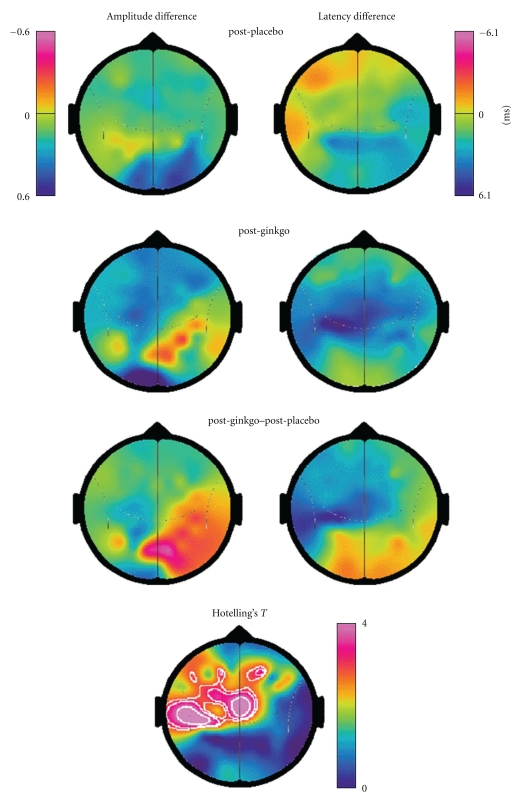
SSVEP topography 1.6 sec into the hold condition. The first row illustrates the SSVEP amplitude difference (left column) and latency difference (right column) for the post-placebo condition with respect to the mean of the post-placebo control task. The second row illustrates the same SSVEP differences for the post-GBE condition while the third row illustrates the differences between the post-GBE and post-Placebo conditions. The third row more clearly illustrates the effects of the GBE on SSVEP topography, in particular an increase in SSVEP latency at left temporal, left frontal, and left prefrontal sites. The single bottom map illustrates the distribution of the square root of the Hotelling's *T* ^2^ parameter with the contours outlining the regions where the *T* parameter exceeds 2.87, 3.19, and 3.92 corresponding to single comparison *P* values of 1%, 0.5%, and 0.1%, respectively.

Using the postplacebo condition to illustrate task-related effects, we noted characteristic SSVEP changes during the intake and hold components of the task that have been previously reported [48], specifically, an SSVEP amplitude reduction during the intake component of the task and an amplitude increase during the hold component. SSVEP latency was transiently reduced during the intake and hold components. The time course of these changes at midline prefrontal (Fz) and occipital sites (Oz) is illustrated in Figures 1 and 2, while the SSVEP topography during the WM or hold component is illustrated in Figure 3.

 The hold component is associated with an SSVEP amplitude increase at parieto-occipital and prefrontal sites (Figure 3, upper left) and an SSVEP latency reduction at left temporal and prefrontal sites.

The major task-related SSVEP amplitude change we observed in the postplacebo condition comprised an increase at occipitoparietal and frontal sites during the hold or WM component of the task. These results are consistent with other observations of a load-dependent increase in the amplitude of the SSVEP during the hold component of a WM task [47, 48]. 

A recent study by Perlstein and colleagues [61] also reported increased SSVEP amplitude in the right dorsolateral prefrontal cortex (DLPFC) during the hold component of a faces WM task. In this case, the amplitude of the SSVEP at the DLPFC during the hold component was correlated with task performance. Specifically, larger SSVEP amplitude was associated with better task performance. Interestingly, an fMRI study of the same faces WM task [62] also showed that the largest Bold response was observed in the DLPFC, the same region responsible for the task-dependent changes in the SSVEP. It should be noted that such load-dependent changes in EEG have also been observed in the alpha frequency range. Specifically, an increased WM load is associated with increased alpha activity or event-related synchronization [63, 64]. 

The major task-related SSVEP latency change we observed in the postplacebo condition comprised a decrease at prefrontal and left temporal sites during the hold or WM component of the task. These results are consistent with other observations of a load-dependent decrease in SSVEP latency at prefrontal sites during the hold component of a WM task [47, 48]. However, the finding of a decrease in SSVEP latency in the left temporal area is a novel finding, which was not previously reported in research by Silberstein and colleagues [48] using a similar WM task.

#### 3.2.2. Ginkgo Effects

SSVEP amplitude and latency topography during the middle of the hold component are illustrated in Figure 3. The differences between the postplacebo (first row) and post-GBE (second row) are illustrated in the third row. GBE effects that are statistically significant include increased amplitude at frontal and parietal sites and increased SSVEP latency at left temporal and left frontal sites.

#### 3.2.3. Effects of GBE on SSVEP Amplitude

During the hold component of the task, the GBE-related occipital SSVEP amplitude increase was more prominent than in the postplacebo condition. The amplitude increase was also more prominent in frontal and prefrontal regions for the GBE condition. While the relationship between this GBE-related SSVEP amplitude increase and the modest improvement in performance we observed is speculative, we suggest a number of observations indicate they are linked. Perlstein and colleagues [61] examined the relationship between performance on a face WM task and SSVEP amplitude. They found that the SSVEP amplitude at prefrontal sites was positively correlated with performance on the task. A study by Van Rooy et al. [65] examined the relationship between IQ and SSVEP amplitude in a spatial WM task. The authors reported that subjects with high full-scale IQ (FSIQ) (range 109–130 on WAIS-R) exhibited higher occipitoparietal SSVEP amplitude compared with the normal FSIQ subjects (range 98–108 on WAIS-R) during the hold component of the spatial WM task. The association of increased SSVEP amplitude with increased performance level and measured IQ is thus consistent with our observation of GBE improving WM performance and SSVEP amplitude. 

The reason that an increase in the amplitude of the SSVEP is associated with increased performance is unclear although two possible explanations suggest themselves. One explanation revolves around the notion that the SSVEP and EEG alpha activity both index a common neural mechanism and that increased alpha activity (event related synchronization) and increased SSVEP represent lower levels of cortical activity or “cortical idling” [66]. In this context, the increased SSVEP is an index of reduced cortical activity and is an indication of reduced cognitive effort. The positive correlation between performance and SSVEP amplitude observed by Perlstein et al. [61] would thus reflect the reduced cognitive effort in the high-performance subjects. Our observation of an increase in SSVEP amplitude could thus be interpreted as an indirect consequence of GBE's enhancement of the efficiency of neural systems participating in the task and thus a reduction in cortical activation. 

Alternatively, the increased SSVEP amplitude and alpha activity may be an indicator of more efficient neural processes responsible for holding information on line. We have previously suggested that corticocortical and corticothalamic loops may contribute to the generation of spontaneous and evoked rhythmic activity in the alpha frequency range [48]. It has also been proposed that these loops may play a crucial role in holding information “on line” in object WM tasks. The observation of increased SSVEP and alpha amplitude with increasing load in a WM task would appear to be consistent with this proposal [48, 61, 63, 64]. In this context, the GBE-related increase in SSVEP amplitude is a consequence of the increased resonant behavior of the corticocortical loops that is in turn related to the increased WM capacity. This suggestion is consistent with the effects of GBE on the ongoing EEG in that GBE has been reported to increase the amplitude of alpha EEG activity [41, 67]. 

The mechanisms responsible for the GBE-induced increase in SSVEP and alpha EEG amplitude is not understood, but a number of lines of evidence point to the importance of cholinergic processes. Animal studies briefly reviewed in the introduction indicate that GBE increases the release of ACh [29] and also increases the density of muscarinic cholinergic receptors in the hippocampus [32]. Drugs that increase the availability of ACh such as cholinesterase inhibitors (ChEIs) cause an increase in EEG alpha amplitude, while those that block cortical cholinergic receptors cause a reduction in EEG alpha activity [68, 69]. Analogous effects have also been observed for the 13 Hz SSVEP. Nicotine, a nonselective cholinergic agonist, increased the amplitude of the 13 Hz SSVEP in a visual vigilance task, while Donepezil, a cholinesterase inhibitor, increased the amplitude of the 13 Hz SSVEP component during the hold component of a spatial WM task [70]. These observations are consistent with the hypothesis that the GBE-related increase in SSVEP amplitude was mediated, in part, by cholinergic processes.

#### 3.2.4. Effects of GBE on SSVEP Latency

In relation to the effect of GBE on SSVEP latency, the most prominent effect was that of an increase in SSVEP latency at left temporal, left prefrontal, and midline frontal sites. By contrast, there was a tendency for GBE to reduce the midline occipital SSVEP latency. We have previously suggested that SSVEP latency changes may index changes in synaptic excitatory and inhibitory processes in the neural networks generating the SSVEP. Specifically, SSVEP latency reductions (faster processing) are a consequence of increased synaptic excitation or reduced inhibition, while SSVEP latency increases (slower processing) indicate reduced synaptic excitation or increased inhibition. This interpretation is consistent with observations that the reaction time in a visual vigilance task (continuous performance task (CPT)) was correlated with frontal SSVEP latency [71]. Subsequent studies examining visual vigilance-related changes in SSVEP latency in schizophrenia [72] and ADHD [47] have also been consistent with this suggestion. 

Our observation of a GBE-related SSVEP latency increase at left temporal, frontal, and left prefrontal sites suggests that GBE is associated with increased levels of synaptic inhibition during the hold component in these regions. This is consistent with our hypothesis that GBE would be associated with increased inhibition at temporal and prefrontal sites and is also consistent with the report of Furey et al. [34, 35] who examined the effects of physostigmine on regional cerebral blood flow in a faces WM task. The group found that physostigmine improved performance (reaction time RT) on the task and that this improvement was correlated with the magnitude of the rCBF decrease at prefrontal and left middle temporal gyrus and left superior temporal sulcus. Our observations of GBE-mediated inhibitory processes at left temporal and prefrontal sites together with PET findings from other laboratories of physostigmine-mediated reductions in brain activity in left temporal and prefrontal sites are consistent with the notion that the cognitive enhancing effects of GBE may in part be mediated by cholinergic mechanisms. Why the activation of such cholinergic mechanisms by physostigmine or GBE should be associated with reductions of left temporal and left prefrontal rCBF and increased inhibition at these sites is unclear. 

One possible explanation that has been suggested is that increased cholinergic activation of the visual cortex enhances attentional processes and thus reduces the need to recruit neural networks in the left temporal and prefrontal cortex thought to contribute to the retrieval or maintenance of the visual image [34, 35]. Alternatively, GBE may enhance WM performance by increasing the cholinergic excitation of inhibitory cells in the frontal and left temporal cortex. Such inhibition needs not to represent reduced information processing by these sites but on the contrary may indicate increased information processing. Cortical GABAergic interneurons receive strong cholinergic innervation that is predominantly excitatory [73]. Such GABAergic cells while only constituting a minority of cortical neurons are thought to play a crucial role in neural information processing [74, 75]. Such cells also play a crucial role in the generation of high-frequency EEG activity or gamma EEG thought to play an important function in the integration of disparate neural networks [76]. The role of the GABAergic system in cognitive information processing has been further emphasized by work demonstrating that the age-related decline in stimulus selectivity of V1 cells located in the striate cortex was caused by the decline of GABAergic cell function [77, 78]. The reduced effectiveness of synaptic inhibitory processes in old age is also suggested by EEG findings pointing to an age-dependent reduction in cortical functional independence [79]. This functional independence is reflected in the dissimilarity of the EEG across scalp sites. Our observation of GBE-mediated left temporal and left prefrontal inhibition may thus indicate increased efficiency of the inhibitory neural system and the consequential enhanced cognitive performance. 

In summary, 14-day administration of GBE was associated with a modest improvement in accuracy in an object WM task and evidence of increased synaptic inhibition at left temporal and prefrontal sites during the hold component of the WM task. We suggest that the improvements in WM task performance are a consequence of enhanced synaptic inhibition associated with GBE, possibly operating via a cholinergic mechanism. These findings provide further evidence for the efficacy of GBE as a treatment for working memory deficits in the elderly.
